# Tris[μ-2,2′-(azinodimethylidyne)diphenolato-κ^4^ *O*,*N*:*N*′,*O*′]diiron(III) tetra­hydro­furan tetra­solvate

**DOI:** 10.1107/S1600536808027438

**Published:** 2008-08-30

**Authors:** Jinglin Wang, Bin Liu, Binsheng Yang

**Affiliations:** a Institute of Molecular Science, Chemical Biology and Molecular Engineering Laboratory of the Education Ministry, Shanxi University, Taiyuan, Shanxi 030006, People’s Republic of China

## Abstract

In the title binuclear iron(III) complex, [Fe_2_(C_14_H_10_N_2_O_2_)_3_]·4C_4_H_8_O or [Fe_2_(salda)_3_]·4THF [H_2_salda = 2,2′-(azinidimethyl­ene)diphenolate and THF is tetra­hydro­furan], the ligand possesses a rotationally flexible single N—N bond. Three dinucleating *O*,*N*:*N*′,*O*′-donor ligands provide three diazine (=N—N=) bridges between the metal ions, yielding a binuclear triple helicate structure with crystallographic *C*_2_ symmetry, the rotation axis bis­ecting one N—N bond.

## Related literature

For related literature, see: Seo *et al.* (2000 ▶); Gao *et al.* (2004 ▶); Oleksi *et al.* (2006 ▶).
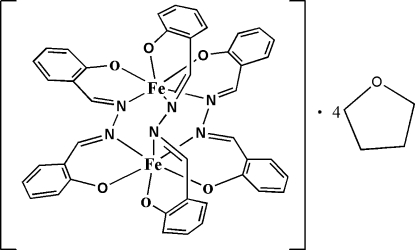


## Experimental

### 

#### Crystal data

[Fe_2_(C_14_H_10_N_2_O_2_)_3_]·4C_4_H_8_O
*M* *_r_* = 1114.84
Orthorhombic, 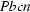

*a* = 15.189 (5) Å
*b* = 19.163 (6) Å
*c* = 18.917 (6) Å
*V* = 5506 (3) Å^3^
*Z* = 4
Mo *K*α radiation
μ = 0.59 mm^−1^
*T* = 293 (2) K
0.40 × 0.40 × 0.20 mm

#### Data collection

Bruker SMART CCD diffractometer
Absorption correction: multi-scan (*SADABS*; Sheldrick, 1996 ▶) *T*_min_ = 0.798, *T*_max_ = 0.891
21523 measured reflections
4856 independent reflections
3520 reflections with *I* > 2σ(*I*)
*R*_int_ = 0.080

#### Refinement

*R*[*F* ^2^ > 2σ(*F* ^2^)] = 0.083
*wR*(*F* ^2^) = 0.222
*S* = 1.08
4856 reflections
343 parameters
H-atom parameters constrained
Δρ_max_ = 0.96 e Å^−3^
Δρ_min_ = −0.42 e Å^−3^

### 

Data collection: *SMART* (Bruker, 2000 ▶); cell refinement: *SAINT* (Bruker, 2000 ▶); data reduction: *SAINT*; program(s) used to solve structure: *SHELXS97* (Sheldrick, 2008 ▶); program(s) used to refine structure: *SHELXL97* (Sheldrick, 2008 ▶); molecular graphics: *SHELXTL* (Sheldrick, 2008 ▶); software used to prepare material for publication: *SHELXTL*.

## Supplementary Material

Crystal structure: contains datablocks I, global. DOI: 10.1107/S1600536808027438/cf2210sup1.cif

Structure factors: contains datablocks I. DOI: 10.1107/S1600536808027438/cf2210Isup2.hkl

Additional supplementary materials:  crystallographic information; 3D view; checkCIF report

## Figures and Tables

**Fig. 1.**
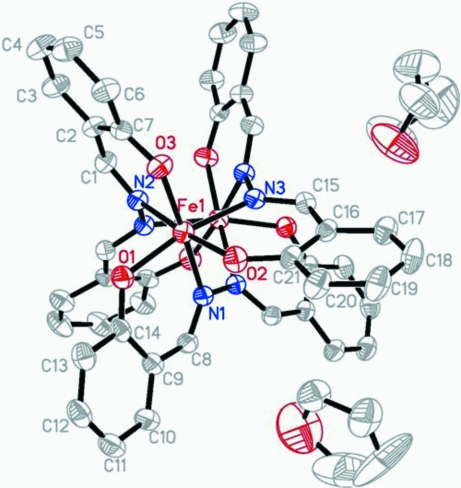
The structure of the title compound, showing the atom-numbering scheme. Displacement ellipsoids are drawn at the 30% probability level. [Symmetry code: A, -x, y, 1/2-z.]

**Fig. 2.**
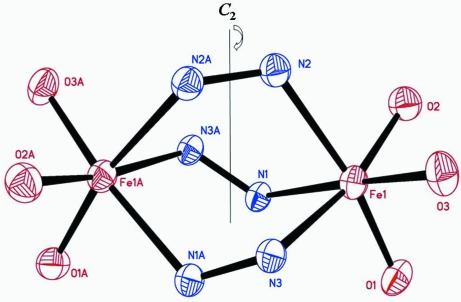
The coordination environment of the Fe(III) atoms. All non-hydrogen atoms are represented by 30% displacement probability ellipsoids. [Symmetry code: A, -x, y, 1/2-z.]

**Fig. 3.**
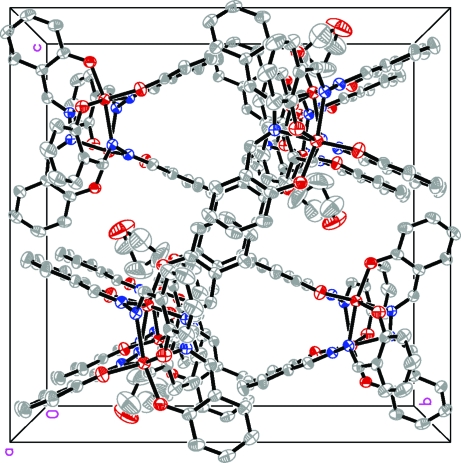
The packing of the title compound.

## supplementary crystallographic information

### Comment

During recent decades, the study of helical coordination complexes or helicates has become an area of intense research activity, due to their intriguing potential applications in enantioselective synthesis and asymmetric catalysis, designing nonlinear optical materials, magnetic materials and porous materials, probing DNA structures, and understanding helical self-organization processes operative in nature (Seo *et al.*, 2000; Gao *et al.*,  2004; Oleksi *et al.*, 2006). In this paper, we report the synthesis and crystal structure of the title complex [Fe_2_(salda)_3_].4THF. Due to the presence of 6 aromatic rings around two Fe(III) ions, the complex displays a large hydrophobic surface and a small diameter (~1.5 nm). These characteristics can be driving forces for the non-covalent recognition of DNA, biological labels and nanoparticle probes by the helicate. The molecular structure of [Fe_2_(salda)_3_].4THF is shown in Fig. 1. In the complex, each of the three ligands coordinates the two metal ions *via* the two phenolate O and the two imine N atoms. Thus, the metal ions are in facial O_3_N_3_ coordination environments and are connected by three diaza (═N—N═) bridges to yield a binuclear triple helicate. The Fe···Fe distance spanned by the triple N—N bridges is 3.9615 (16) Å. The three Fe—N—N—Fe torsion angles are -48.1 (5)° and two symmetry-equivalent at -67.6 (3)°; The complex has C_2_ symmetry with a 2-fold rotation axis through the midpoints of Fe1···Fe1A and N2—N2A (symmetry code as in Fig. 2). A packing diagram is shown in Fig. 3.

### Experimental

The ligand H_2_salda was prepared by reacting hydrazine with 2 equiv of salicylaldehyde followed by recrystallization from methanol. To a solution of 360 mg (1.5 mmol) of H_2_salda dissolved in 30 ml of methanol was added a solution of 3% (g/100 ml) sodium methoxide in 0.4 ml methanol. FeCl_3._H_2_O (270 mg, 1 mmol) in methanol (15 ml) was added dropwise to the resulting brownish-black solution. The reaction mixture was stirred and refluxed on a water bath for 2 h. The resulting precipitate was filtered off, and washed successively with methanol and THF prior to drying *in vacuo*. The dried powder was dissolved in THF and crystals suitable for X-ray analysis were obtained after two weeks at room temperature.

### Refinement

The H atoms were treated as riding atoms, with C—H = 0.93–0.97 Å, *U*_iso_(H) = 1.2*U*_eq_(C).

### Crystal data

**Table:**

[Fe_2_(C_14_H_10_N_2_O_2_)_3_]·4C_4_H_8_O	*D*_x_ = 1.345 Mg m^−^^3^
*M**_r_* = 1114.84	Mo *K*α radiation λ = 0.71073 Å
Orthorhombic, *P**b**c**n*	Cell parameters from 30326 reflections
*a* = 15.189 (5) Å	θ = 1.7–25.0º
*b* = 19.163 (6) Å	µ = 0.59 mm^−^^1^
*c* = 18.917 (6) Å	*T* = 293 (2) K
*V* = 5506 (3) Å^3^	Block, black
*Z* = 4	0.40 × 0.40 × 0.20 mm
*F*_000_ = 2336	

### Data collection

**Table:**

Bruker SMART CCD diffractometer	4856 independent reflections
Radiation source: fine-focus sealed tube	3520 reflections with *I* > 2σ(*I*)
Monochromator: graphite	*R*_int_ = 0.080
*T* = 293(2) K	θ_max_ = 25.0º
φ and ω scans	θ_min_ = 1.7º
Absorption correction: multi-scan(SADABS; Sheldrick, 1996)	*h* = −18→11
*T*_min_ = 0.798, *T*_max_ = 0.891	*k* = −21→22
21523 measured reflections	*l* = −21→22

### Refinement

**Table:**

Refinement on *F*^2^	Secondary atom site location: difference Fourier map
Least-squares matrix: full	Hydrogen site location: inferred from neighbouring sites
*R*[*F*^2^ > 2σ(*F*^2^)] = 0.083	H-atom parameters constrained
*wR*(*F*^2^) = 0.222	*w* = 1/[σ^2^(*F*_o_^2^) + (0.1154*P*)^2^ + 5.1454*P*] where *P* = (*F*_o_^2^ + 2*F*_c_^2^)/3
*S* = 1.09	(Δ/σ)_max_ < 0.001
4856 reflections	Δρ_max_ = 0.96 e Å^−^^3^
343 parameters	Δρ_min_ = −0.42 e Å^−^^3^
Primary atom site location: structure-invariant direct methods	Extinction correction: none

### Special details

**Table:**

Geometry. All e.s.d.'s (except the e.s.d. in the dihedral angle between two l.s. planes) are estimated using the full covariance matrix. The cell e.s.d.'s are taken into account individually in the estimation of e.s.d.'s in distances, angles and torsion angles; correlations between e.s.d.'s in cell parameters are only used when they are defined by crystal symmetry. An approximate (isotropic) treatment of cell e.s.d.'s is used for estimating e.s.d.'s involving l.s. planes.
Refinement. Refinement of *F*^2^ against ALL reflections. The weighted *R*-factor *wR* and goodness of fit *S* are based on *F*^2^, conventional *R*-factors *R* are based on *F*, with *F* set to zero for negative *F*^2^. The threshold expression of *F*^2^ > σ(*F*^2^) is used only for calculating *R*-factors(gt) *etc*. and is not relevant to the choice of reflections for refinement. *R*-factors based on *F*^2^ are statistically about twice as large as those based on *F*, and *R*- factors based on ALL data will be even larger.

### Fractional atomic coordinates and isotropic or equivalent isotropic displacement parameters (Å^2^)

**Table:**

	*x*	*y*	*z*	*U*_iso_*/*U*_eq_	
C22	0.140 (2)	0.6626 (11)	0.2989 (14)	0.317 (18)	
H22A	0.1376	0.6984	0.2628	0.380*	
H22B	0.0997	0.6757	0.3364	0.380*	
Fe1	0.10583 (4)	0.30805 (3)	0.18882 (3)	0.0431 (3)	
O2	0.0996 (2)	0.34432 (18)	0.09508 (17)	0.0530 (9)	
O3	0.2127 (2)	0.35565 (19)	0.21251 (18)	0.0560 (9)	
O1	0.1549 (2)	0.21832 (18)	0.16629 (19)	0.0539 (9)	
N2	0.0242 (2)	0.39989 (19)	0.21748 (19)	0.0423 (9)	
N1	−0.0194 (2)	0.2536 (2)	0.17748 (18)	0.0410 (9)	
N3	0.0962 (2)	0.2897 (2)	0.3007 (2)	0.0416 (9)	
C7	−0.0316 (3)	0.1927 (2)	0.1519 (3)	0.0478 (11)	
H7	−0.0896	0.1779	0.1467	0.057*	
C6	0.0360 (3)	0.1452 (2)	0.1305 (3)	0.0473 (11)	
C5	0.0111 (4)	0.0804 (3)	0.1022 (3)	0.0631 (15)	
H5	−0.0485	0.0704	0.0975	0.076*	
C4	0.0709 (4)	0.0318 (3)	0.0814 (3)	0.0728 (17)	
H4	0.0528	−0.0110	0.0631	0.087*	
C3	0.1602 (4)	0.0479 (3)	0.0882 (3)	0.0688 (16)	
H3	0.2021	0.0153	0.0742	0.083*	
C2	0.1870 (4)	0.1102 (3)	0.1147 (3)	0.0604 (14)	
H2	0.2469	0.1197	0.1176	0.072*	
C1	0.1264 (3)	0.1605 (2)	0.1380 (2)	0.0461 (11)	
C14	0.0218 (3)	0.4552 (3)	0.1792 (2)	0.0462 (11)	
H14	−0.0117	0.4923	0.1961	0.055*	
C13	0.0658 (3)	0.4651 (3)	0.1129 (2)	0.0464 (11)	
C12	0.0676 (4)	0.5322 (3)	0.0848 (3)	0.0568 (13)	
H12	0.0416	0.5684	0.1100	0.068*	
C11	0.1067 (4)	0.5466 (3)	0.0209 (3)	0.0662 (16)	
H11	0.1088	0.5920	0.0036	0.079*	
C10	0.1426 (4)	0.4923 (3)	−0.0169 (3)	0.0673 (16)	
H10	0.1685	0.5013	−0.0605	0.081*	
C9	0.1413 (4)	0.4253 (3)	0.0082 (3)	0.0619 (14)	
H9	0.1664	0.3899	−0.0186	0.074*	
C8	0.1028 (3)	0.4088 (3)	0.0735 (2)	0.0481 (12)	
C21	0.1480 (3)	0.3112 (2)	0.3498 (2)	0.0453 (11)	
H21	0.1329	0.3008	0.3963	0.054*	
C20	0.2278 (3)	0.3502 (2)	0.3381 (3)	0.0484 (12)	
C19	0.2779 (4)	0.3683 (3)	0.3974 (3)	0.0654 (15)	
H19	0.2580	0.3560	0.4422	0.078*	
C18	0.3563 (4)	0.4040 (4)	0.3904 (4)	0.083 (2)	
H18	0.3898	0.4153	0.4300	0.100*	
C17	0.3842 (4)	0.4227 (4)	0.3237 (4)	0.080 (2)	
H17	0.4367	0.4472	0.3188	0.096*	
C16	0.3368 (4)	0.4064 (3)	0.2649 (3)	0.0660 (16)	
H16	0.3577	0.4196	0.2206	0.079*	
C15	0.2565 (3)	0.3698 (2)	0.2702 (3)	0.0490 (12)	
C23	0.2248 (11)	0.6588 (9)	0.3259 (10)	0.250 (13)	
H23A	0.2241	0.6661	0.3766	0.300*	
H23B	0.2616	0.6944	0.3046	0.300*	
C24	0.2577 (6)	0.5921 (6)	0.3100 (5)	0.125 (3)	
H24A	0.2754	0.5683	0.3529	0.149*	
H24B	0.3084	0.5957	0.2790	0.149*	
C25	0.1866 (5)	0.5537 (5)	0.2749 (5)	0.103 (2)	
H25A	0.2058	0.5390	0.2284	0.123*	
H25B	0.1721	0.5124	0.3021	0.123*	
C26	0.0389 (9)	0.2553 (8)	0.5461 (8)	0.195 (6)	
H26A	0.0013	0.2226	0.5212	0.234*	
H26B	0.0078	0.2988	0.5537	0.234*	
O5	0.1208 (6)	0.2653 (5)	0.5114 (4)	0.182 (4)	
C27	0.074 (2)	0.2259 (13)	0.6122 (9)	0.319 (16)	
H27A	0.1107	0.2581	0.6382	0.382*	
H27B	0.0281	0.2068	0.6427	0.382*	
O4	0.1119 (6)	0.5964 (7)	0.2687 (6)	0.210 (4)	
C29	0.1815 (9)	0.2172 (7)	0.5369 (8)	0.176 (5)	
H29A	0.2243	0.2388	0.5681	0.212*	
H29B	0.2118	0.1931	0.4989	0.212*	
C28	0.123 (2)	0.1724 (9)	0.5741 (11)	0.336 (19)	
H28A	0.1541	0.1412	0.6058	0.403*	
H28B	0.0860	0.1456	0.5424	0.403*	

### Atomic displacement parameters (Å^2^)

**Table:**

	*U*^11^	*U*^22^	*U*^33^	*U*^12^	*U*^13^	*U*^23^
C22	0.46 (4)	0.135 (14)	0.35 (3)	0.10 (2)	0.18 (3)	0.072 (18)
Fe1	0.0335 (4)	0.0488 (4)	0.0470 (4)	−0.0021 (3)	0.0011 (3)	−0.0014 (3)
O2	0.057 (2)	0.057 (2)	0.0457 (18)	0.0009 (17)	0.0023 (16)	−0.0001 (16)
O3	0.042 (2)	0.070 (2)	0.055 (2)	−0.0128 (17)	0.0025 (17)	0.0021 (17)
O1	0.0350 (18)	0.054 (2)	0.073 (2)	0.0036 (16)	−0.0004 (17)	−0.0111 (18)
N2	0.037 (2)	0.046 (2)	0.044 (2)	−0.0020 (17)	0.0004 (17)	0.0001 (17)
N1	0.031 (2)	0.046 (2)	0.046 (2)	0.0018 (17)	0.0020 (16)	−0.0027 (17)
N3	0.031 (2)	0.045 (2)	0.048 (2)	−0.0015 (16)	0.0011 (17)	0.0037 (17)
C7	0.037 (3)	0.052 (3)	0.055 (3)	−0.007 (2)	0.003 (2)	−0.004 (2)
C6	0.044 (3)	0.044 (3)	0.054 (3)	−0.003 (2)	0.009 (2)	−0.003 (2)
C5	0.055 (3)	0.051 (3)	0.083 (4)	−0.006 (3)	0.012 (3)	−0.007 (3)
C4	0.074 (4)	0.054 (3)	0.091 (4)	−0.003 (3)	0.015 (3)	−0.018 (3)
C3	0.065 (4)	0.058 (3)	0.083 (4)	0.013 (3)	0.021 (3)	−0.008 (3)
C2	0.048 (3)	0.057 (3)	0.076 (4)	0.006 (3)	0.010 (3)	−0.005 (3)
C1	0.048 (3)	0.044 (3)	0.047 (3)	0.005 (2)	0.004 (2)	0.004 (2)
C14	0.041 (3)	0.046 (3)	0.052 (3)	0.003 (2)	−0.005 (2)	−0.001 (2)
C13	0.037 (3)	0.055 (3)	0.047 (3)	−0.003 (2)	−0.003 (2)	0.006 (2)
C12	0.052 (3)	0.057 (3)	0.061 (3)	−0.002 (3)	−0.003 (3)	0.010 (3)
C11	0.061 (4)	0.072 (4)	0.066 (4)	−0.010 (3)	−0.007 (3)	0.024 (3)
C10	0.061 (4)	0.092 (5)	0.049 (3)	−0.014 (3)	0.000 (3)	0.016 (3)
C9	0.052 (3)	0.083 (4)	0.050 (3)	−0.004 (3)	0.002 (3)	0.002 (3)
C8	0.036 (3)	0.064 (3)	0.044 (3)	−0.005 (2)	−0.009 (2)	0.006 (2)
C21	0.040 (3)	0.054 (3)	0.042 (2)	0.007 (2)	0.001 (2)	−0.002 (2)
C20	0.038 (3)	0.049 (3)	0.058 (3)	−0.001 (2)	−0.005 (2)	−0.009 (2)
C19	0.049 (3)	0.078 (4)	0.069 (4)	−0.001 (3)	0.000 (3)	−0.016 (3)
C18	0.054 (4)	0.115 (5)	0.080 (4)	−0.020 (4)	−0.015 (3)	−0.032 (4)
C17	0.048 (3)	0.103 (5)	0.090 (5)	−0.028 (3)	−0.002 (3)	−0.023 (4)
C16	0.044 (3)	0.077 (4)	0.077 (4)	−0.020 (3)	0.009 (3)	−0.011 (3)
C15	0.038 (3)	0.050 (3)	0.059 (3)	−0.002 (2)	0.000 (2)	−0.011 (2)
C23	0.224 (17)	0.188 (15)	0.34 (2)	−0.160 (15)	0.196 (18)	−0.177 (16)
C24	0.085 (6)	0.165 (9)	0.124 (7)	−0.052 (6)	0.006 (5)	0.001 (6)
C25	0.073 (5)	0.115 (6)	0.120 (6)	−0.013 (5)	0.002 (5)	0.006 (5)
C26	0.164 (12)	0.233 (15)	0.187 (13)	0.079 (11)	0.089 (10)	0.075 (11)
O5	0.163 (7)	0.243 (9)	0.139 (6)	0.081 (7)	0.057 (5)	0.088 (6)
C27	0.52 (4)	0.32 (3)	0.115 (11)	−0.11 (3)	0.108 (18)	−0.027 (15)
O4	0.122 (7)	0.246 (12)	0.264 (12)	0.049 (8)	−0.028 (7)	−0.002 (10)
C29	0.148 (11)	0.176 (11)	0.204 (12)	0.071 (10)	−0.064 (10)	−0.009 (10)
C28	0.62 (5)	0.151 (14)	0.24 (2)	−0.18 (2)	−0.12 (3)	0.118 (15)

### Geometric parameters (Å, °)

**Table:**

C22—C23	1.39 (3)	C10—C9	1.368 (8)
C22—O4	1.46 (3)	C10—H10	0.930
C22—H22A	0.970	C9—C8	1.402 (7)
C22—H22B	0.970	C9—H9	0.930
Fe1—O2	1.907 (3)	C21—C20	1.441 (7)
Fe1—O3	1.916 (3)	C21—H21	0.930
Fe1—O1	1.922 (3)	C20—C19	1.399 (7)
Fe1—N3	2.150 (4)	C20—C15	1.406 (7)
Fe1—N1	2.180 (4)	C19—C18	1.381 (8)
Fe1—N2	2.220 (4)	C19—H19	0.930
O2—C8	1.303 (6)	C18—C17	1.378 (9)
O3—C15	1.306 (6)	C18—H18	0.930
O1—C1	1.304 (6)	C17—C16	1.362 (8)
N2—C14	1.285 (6)	C17—H17	0.930
N2—N2^i^	1.433 (7)	C16—C15	1.411 (7)
N1—C7	1.276 (6)	C16—H16	0.930
N1—N3^i^	1.419 (5)	C23—C24	1.406 (18)
N3—C21	1.286 (6)	C23—H23A	0.970
N3—N1^i^	1.419 (5)	C23—H23B	0.970
C7—C6	1.430 (7)	C24—C25	1.465 (11)
C7—H7	0.930	C24—H24A	0.970
C6—C5	1.403 (7)	C24—H24B	0.970
C6—C1	1.411 (7)	C25—O4	1.403 (12)
C5—C4	1.360 (8)	C25—H25A	0.970
C5—H5	0.930	C25—H25B	0.970
C4—C3	1.398 (9)	C26—O5	1.420 (13)
C4—H4	0.930	C26—C27	1.47 (2)
C3—C2	1.357 (8)	C26—H26A	0.970
C3—H3	0.930	C26—H26B	0.970
C2—C1	1.405 (7)	O5—C29	1.389 (12)
C2—H2	0.930	C27—C28	1.46 (3)
C14—C13	1.433 (7)	C27—H27A	0.970
C14—H14	0.930	C27—H27B	0.970
C13—C12	1.393 (7)	C29—C28	1.42 (2)
C13—C8	1.426 (7)	C29—H29A	0.970
C12—C11	1.375 (8)	C29—H29B	0.970
C12—H12	0.930	C28—H28A	0.970
C11—C10	1.375 (9)	C28—H28B	0.970
C11—H11	0.930		
			
C23—C22—O4	111.8 (16)	C10—C9—H9	119.2
C23—C22—H22A	109.3	C8—C9—H9	119.2
O4—C22—H22A	109.3	O2—C8—C9	120.3 (5)
C23—C22—H22B	109.3	O2—C8—C13	122.6 (4)
O4—C22—H22B	109.3	C9—C8—C13	117.0 (5)
H22A—C22—H22B	107.9	N3—C21—C20	124.7 (4)
O2—Fe1—O3	94.95 (15)	N3—C21—H21	117.7
O2—Fe1—O1	98.00 (15)	C20—C21—H21	117.7
O3—Fe1—O1	98.58 (15)	C19—C20—C15	119.9 (5)
O2—Fe1—N3	166.26 (15)	C19—C20—C21	117.5 (5)
O3—Fe1—N3	84.55 (14)	C15—C20—C21	122.6 (4)
O1—Fe1—N3	95.65 (15)	C18—C19—C20	121.0 (6)
O2—Fe1—N1	92.27 (14)	C18—C19—H19	119.5
O3—Fe1—N1	172.09 (14)	C20—C19—H19	119.5
O1—Fe1—N1	83.57 (14)	C17—C18—C19	118.8 (6)
N3—Fe1—N1	87.67 (13)	C17—C18—H18	120.6
O2—Fe1—N2	84.86 (14)	C19—C18—H18	120.6
O3—Fe1—N2	92.21 (15)	C16—C17—C18	121.8 (6)
O1—Fe1—N2	168.52 (14)	C16—C17—H17	119.1
N3—Fe1—N2	81.44 (14)	C18—C17—H17	119.1
N1—Fe1—N2	85.21 (14)	C17—C16—C15	120.8 (6)
C8—O2—Fe1	129.4 (3)	C17—C16—H16	119.6
C15—O3—Fe1	136.5 (3)	C15—C16—H16	119.6
C1—O1—Fe1	136.2 (3)	O3—C15—C20	123.4 (4)
C14—N2—N2^i^	118.0 (3)	O3—C15—C16	118.9 (5)
C14—N2—Fe1	122.2 (3)	C20—C15—C16	117.8 (5)
N2^i^—N2—Fe1	119.75 (17)	C22—C23—C24	107.4 (14)
C7—N1—N3^i^	115.9 (4)	C22—C23—H23A	110.2
C7—N1—Fe1	127.0 (3)	C24—C23—H23A	110.2
N3^i^—N1—Fe1	117.1 (3)	C22—C23—H23B	110.2
C21—N3—N1^i^	116.7 (4)	C24—C23—H23B	110.2
C21—N3—Fe1	128.2 (3)	H23A—C23—H23B	108.5
N1^i^—N3—Fe1	115.0 (3)	C23—C24—C25	106.9 (10)
N1—C7—C6	125.8 (5)	C23—C24—H24A	110.3
N1—C7—H7	117.1	C25—C24—H24A	110.3
C6—C7—H7	117.1	C23—C24—H24B	110.3
C5—C6—C1	119.0 (5)	C25—C24—H24B	110.3
C5—C6—C7	118.5 (5)	H24A—C24—H24B	108.6
C1—C6—C7	122.5 (4)	O4—C25—C24	110.0 (9)
C4—C5—C6	122.5 (5)	O4—C25—H25A	109.7
C4—C5—H5	118.7	C24—C25—H25A	109.7
C6—C5—H5	118.7	O4—C25—H25B	109.7
C5—C4—C3	118.0 (5)	C24—C25—H25B	109.7
C5—C4—H4	121.0	H25A—C25—H25B	108.2
C3—C4—H4	121.0	O5—C26—C27	97.6 (14)
C2—C3—C4	121.3 (5)	O5—C26—H26A	112.2
C2—C3—H3	119.3	C27—C26—H26A	112.2
C4—C3—H3	119.3	O5—C26—H26B	112.2
C3—C2—C1	121.6 (5)	C27—C26—H26B	112.2
C3—C2—H2	119.2	H26A—C26—H26B	109.8
C1—C2—H2	119.2	C29—O5—C26	109.3 (10)
O1—C1—C2	119.6 (5)	C28—C27—C26	91.9 (13)
O1—C1—C6	122.8 (4)	C28—C27—H27A	113.3
C2—C1—C6	117.6 (5)	C26—C27—H27A	113.3
N2—C14—C13	126.0 (5)	C28—C27—H27B	113.3
N2—C14—H14	117.0	C26—C27—H27B	113.3
C13—C14—H14	117.0	H27A—C27—H27B	110.6
C12—C13—C8	119.3 (5)	C25—O4—C22	103.8 (14)
C12—C13—C14	117.8 (5)	O5—C29—C28	99.1 (14)
C8—C13—C14	122.8 (4)	O5—C29—H29A	112.0
C11—C12—C13	122.1 (5)	C28—C29—H29A	112.0
C11—C12—H12	119.0	O5—C29—H29B	112.0
C13—C12—H12	119.0	C28—C29—H29B	112.0
C12—C11—C10	118.4 (5)	H29A—C29—H29B	109.6
C12—C11—H11	120.8	C29—C28—C27	98.2 (14)
C10—C11—H11	120.8	C29—C28—H28A	112.1
C9—C10—C11	121.6 (5)	C27—C28—H28A	112.1
C9—C10—H10	119.2	C29—C28—H28B	112.1
C11—C10—H10	119.2	C27—C28—H28B	112.1
C10—C9—C8	121.5 (6)	H28A—C28—H28B	109.8
			
O3—Fe1—O2—C8	−52.8 (4)	C4—C3—C2—C1	1.4 (9)
O1—Fe1—O2—C8	−152.2 (4)	Fe1—O1—C1—C2	165.2 (4)
N3—Fe1—O2—C8	34.5 (9)	Fe1—O1—C1—C6	−15.9 (7)
N1—Fe1—O2—C8	123.9 (4)	C3—C2—C1—O1	176.7 (5)
N2—Fe1—O2—C8	39.0 (4)	C3—C2—C1—C6	−2.2 (8)
O2—Fe1—O3—C15	165.6 (5)	C5—C6—C1—O1	−177.2 (5)
O1—Fe1—O3—C15	−95.5 (5)	C7—C6—C1—O1	1.5 (7)
N3—Fe1—O3—C15	−0.6 (5)	C5—C6—C1—C2	1.6 (7)
N1—Fe1—O3—C15	9.8 (14)	C7—C6—C1—C2	−179.7 (5)
N2—Fe1—O3—C15	80.6 (5)	N2^i^—N2—C14—C13	179.9 (5)
O2—Fe1—O1—C1	−73.9 (5)	Fe1—N2—C14—C13	3.2 (7)
O3—Fe1—O1—C1	−170.2 (4)	N2—C14—C13—C12	−169.5 (5)
N3—Fe1—O1—C1	104.5 (5)	N2—C14—C13—C8	14.1 (8)
N1—Fe1—O1—C1	17.5 (4)	C8—C13—C12—C11	−2.2 (8)
N2—Fe1—O1—C1	29.8 (10)	C14—C13—C12—C11	−178.7 (5)
O2—Fe1—N2—C14	−22.5 (4)	C13—C12—C11—C10	1.9 (8)
O3—Fe1—N2—C14	72.3 (4)	C12—C11—C10—C9	−1.0 (9)
O1—Fe1—N2—C14	−127.5 (7)	C11—C10—C9—C8	0.3 (9)
N3—Fe1—N2—C14	156.4 (4)	Fe1—O2—C8—C9	147.1 (4)
N1—Fe1—N2—C14	−115.2 (4)	Fe1—O2—C8—C13	−34.9 (6)
O2—Fe1—N2—N2^i^	160.9 (4)	C10—C9—C8—O2	177.6 (5)
O3—Fe1—N2—N2^i^	−104.4 (4)	C10—C9—C8—C13	−0.5 (7)
O1—Fe1—N2—N2^i^	55.8 (9)	C12—C13—C8—O2	−176.6 (4)
N3—Fe1—N2—N2^i^	−20.2 (4)	C14—C13—C8—O2	−0.3 (7)
N1—Fe1—N2—N2^i^	68.2 (4)	C12—C13—C8—C9	1.4 (7)
O2—Fe1—N1—C7	86.4 (4)	C14—C13—C8—C9	177.8 (5)
O3—Fe1—N1—C7	−117.7 (11)	N1^i^—N3—C21—C20	−178.5 (4)
O1—Fe1—N1—C7	−11.4 (4)	Fe1—N3—C21—C20	−3.4 (7)
N3—Fe1—N1—C7	−107.4 (4)	N3—C21—C20—C19	−178.6 (5)
N2—Fe1—N1—C7	171.0 (4)	N3—C21—C20—C15	1.1 (7)
O2—Fe1—N1—N3^i^	−92.2 (3)	C15—C20—C19—C18	−1.3 (8)
O3—Fe1—N1—N3^i^	63.7 (12)	C21—C20—C19—C18	178.3 (5)
O1—Fe1—N1—N3^i^	170.0 (3)	C20—C19—C18—C17	1.0 (10)
N3—Fe1—N1—N3^i^	74.1 (3)	C19—C18—C17—C16	−0.5 (11)
N2—Fe1—N1—N3^i^	−7.5 (3)	C18—C17—C16—C15	0.4 (11)
O2—Fe1—N3—C21	−85.7 (7)	Fe1—O3—C15—C20	−1.1 (8)
O3—Fe1—N3—C21	2.8 (4)	Fe1—O3—C15—C16	178.7 (4)
O1—Fe1—N3—C21	100.9 (4)	C19—C20—C15—O3	−179.0 (5)
N1—Fe1—N3—C21	−175.8 (4)	C21—C20—C15—O3	1.3 (7)
N2—Fe1—N3—C21	−90.2 (4)	C19—C20—C15—C16	1.2 (7)
O2—Fe1—N3—N1^i^	89.4 (7)	C21—C20—C15—C16	−178.5 (5)
O3—Fe1—N3—N1^i^	177.9 (3)	C17—C16—C15—O3	179.5 (6)
O1—Fe1—N3—N1^i^	−84.0 (3)	C17—C16—C15—C20	−0.7 (8)
N1—Fe1—N3—N1^i^	−0.7 (3)	O4—C22—C23—C24	4(2)
N2—Fe1—N3—N1^i^	84.9 (3)	C22—C23—C24—C25	−2.9 (19)
N3^i^—N1—C7—C6	−176.0 (4)	C23—C24—C25—O4	0.7 (13)
Fe1—N1—C7—C6	5.4 (7)	C27—C26—O5—C29	27.3 (18)
N1—C7—C6—C5	−179.0 (5)	O5—C26—C27—C28	−54 (2)
N1—C7—C6—C1	2.3 (8)	C24—C25—O4—C22	1.6 (15)
C1—C6—C5—C4	−0.3 (8)	C23—C22—O4—C25	−4(2)
C7—C6—C5—C4	−179.1 (5)	C26—O5—C29—C28	12.4 (18)
C6—C5—C4—C3	−0.5 (9)	O5—C29—C28—C27	−48 (2)
C5—C4—C3—C2	0.0 (9)	C26—C27—C28—C29	64 (2)

Symmetry codes: (i) −*x*, *y*, −*z*+1/2.
